# Effect of COVID-19 Quarantine on the Sleep Quality and the Depressive Symptom Levels of University Students in Jordan During the Spring of 2020

**DOI:** 10.3389/fpsyt.2021.605676

**Published:** 2021-02-16

**Authors:** Heba Saadeh, Maha Saadeh, Wesam Almobaideen, Assem Al Refaei, Nour Shewaikani, Reem Qadan Al Fayez, Hamzah Khawaldah, Sobuh Abu-Shanab, Maysa Al-Hussaini

**Affiliations:** ^1^Computer Science Department, King Abdullah II School of Information Technology Faculty, The University of Jordan, Amman, Jordan; ^2^Computer Engineering and Informatics, Middlesex University Dubai, Dubai, United Arab Emirates; ^3^Electrical Engineering and Computing Sciences, Rochester Institute of Technology, Dubai, United Arab Emirates; ^4^School of Medicine, The University of Jordan, Amman, Jordan; ^5^Computer Information System Department, King Abdullah II School of Information Technology Faculty, The University of Jordan, Amman, Jordan; ^6^Geography Department, School of Arts, The University of Jordan, Amman, Jordan; ^7^Psychosocial Program, King Hussein Cancer Center, Amman, Jordan; ^8^Department of Pathology and Laboratory Medicine, King Hussein Cancer Center, Amman, Jordan; ^9^Human Research Protection Program Office, King Hussein Cancer Center, Amman, Jordan

**Keywords:** University students, mental health, COVID-19 quarantine, PSQI, CES-D

## Abstract

**Objectives:** This study was designed to assess the effect of COVID-19 home quarantine and its lifestyle challenges on the sleep quality and mental health of a large sample of undergraduate University students in Jordan. It is the first study applied to the Jordanian population. The aim was to investigate how quarantine for several weeks changed the students' habits and affected their mental health.

**Methods:** A cross-sectional study was conducted using a random representative sample of 6,157 undergraduate students (mean age 19.79 ± 1.67 years, males 28.7%) from the University of Jordan through voluntarily filling an online questionnaire. The Pittsburgh Sleep Quality Index (PSQI) and the Center for Epidemiologic Studies-Depression Scale (CES-D) were used to assess sleep quality and depressive symptoms, respectively.

**Results:** The PSQI mean score for the study participants was 8.1 ± 3.6. The sleep quality of three-quarters of the participants was negatively affected by the extended quarantine. Nearly half of the participants reported poor sleep quality. The prevalence of poor sleep quality among participants was 76% (males: 71.5% and females: 77.8%). Similarly, the prevalence of the depressive symptoms was 71% (34% for moderate and 37% for high depressive symptoms), with females showing higher prevalence than males. The overall mean CES-D score for the group with low depressive symptoms is 9.3, for the moderate group is 19.8, while it is 34.3 for the high depressive symptoms group. More than half of the students (62.5%) reported that the quarantine had a negative effect on their mental health. Finally, females, smokers, and students with decreased income levels during the extended quarantine were the common exposures that are significantly associated with a higher risk of developing sleep disturbances and depressive symptoms.

**Conclusions:** Mass and extended quarantine succeeded in controlling the spread of the COVID-19 virus; however, it comes with a high cost of potential psychological impacts. Most of the students reported that they suffer from sleeping disorders and had a degree of depressive symptoms. Officials should provide psychological support and clear guidance to help the general public to reduce these potential effects and overcome the quarantine period with minimum negative impacts.

## Introduction

In early December of 2019, the novel Severe Acute Respiratory Syndrome Coronavirus 2 (SARS-CoV-2), known later as COVID-19, emerged in Wuhan city of China (1). As of 20th of December 2020, COVID-19 affected more than 200 countries, with more than 77 million cases and a death toll that is nearly two million globally (2). This respiratory pandemic is highly contagious, and containment strategies include quarantine, lockdown, isolation, travel bans, country-wide closure, social distancing, personal hygiene, and face-mask mandating were applied by many countries (3). These stringent measures helped in controlling the virus spread. Many countries had applied quarantine or stay at home procedures from the detection of early cases; it aims to restrict people's movement and reduce their social mixing (4). Even though quarantine limits the spread of COVID-19 and other infectious diseases (5–9), its psychological effect, along with its social, economic, and physiological impacts, should not be neglected (10–14).

Having sufficient sleep at night plays an essential role in the efficiency of accomplishing everyday tasks and having good mental abilities (15). Globally, inadequate sleep is considered a public health epidemic, being linked to 7 of the 15 leading causes of death in the U.S. (16). A study among Canadians reported that poor sleep quality with short sleep duration was prevalent, as 43% of men and 55% of women had a disturbance in sleeping or staying asleep (17). Another study in Ethiopia reported poor sleep quality among 65.4% of the participants (18). Furthermore, in Saudi Arabia, a study conducted on a sample of health care workers revealed that 42.3% suffer from poor sleep quality (19).

Several studies assess the sleep quality amid the COVID-19 pandemic. In France, deteriorated sleep quality during the current quarantine was reported by 47% of the study sample (20). 57.1% of Italians who participated in an online questionnaire suffered from decreased sleep quality (21). Moreover, during the 2 weeks of quarantine in February in China, the sleeping disorders were significantly increased in the age group of 18–24 years (22). Likewise, a study in Greece showed that although the quantity of sleeping hours increased in 66.3% of the study participants, the sleep quality decreases to 43% (23). Furthermore, more than half of the Spanish participants in a study reported a change in their sleeping habits due to quarantine (24).

Depression is a widespread mental disorder that affects millions of people worldwide, and it is the leading cause of disability (25). Persistent negative thoughts, feeling down, lack of energy, losing interest in joyful activities, sleep disturbance, and many more are among the common symptoms of depression (25). This long-lasting pessimistic mood may lead to suicidal thoughts (26, 27). Many people suffering from depressive symptoms tend to escape real-life and dealing with surrounding family, friends, and colleagues into social media looking for comforts and relief in positive comments and news, which is reflected in their high usage of their smart devices; like smartphones, tablets, iPad, and other devices (28–32). Globally, 40.5% (31.7–49.2%) of the disability-adjusted years of life caused by depressive disorders, with a 4.7% (4.4–5.0%) global prevalence of major depressive disorders and an annual incidence of 3.0% (2.4–3.8%) (33, 34). Regionally, researchers in the Middle East and North Africa regions had evaluated depressive symptoms rates ranging from around 13 to 29%, with women and University students having higher rates, among others (35). Another study reported depressive symptoms among University students in Oman and Egypt as 27.7 and 60.8%, respectively (36, 37). In Jordan, around 74% showed a degree of depressive symptoms among school and University students (38, 39).

The fear of the current pandemic and its consequences, especially on the economy, caused a depression that sometimes leads to suicidal incidents (40–42). The extended quarantine and disturbance of everyday life routine increase the anxiety and depression levels. In Southwestern China, Lei et al. reported significant differences in the prevalence of anxiety and depressive symptoms among the public affected by quarantine (12.9 and 22.4%, respectively) and those unaffected (6.7 and 11.9%, respectively) during the COVID-19 pandemic (43). Similarly, after the stay-at-home order was issued, Spanish researchers identified higher levels of depressive symptoms in northern Spain, specifically among younger individuals with chronic diseases (44). These results are in line with the 2003 SARS outbreak findings in which the sample group that showed the highest levels of depression symptoms were quarantined during the outbreak (45).

Treating and taking care of COVID-19 infected patients, in addition to protecting others from catching the virus, are the priority for most countries worldwide. However, COVID-19 has psychological stress impact on non-infected members, which may last longer than the pandemic's actual time. Understanding the level and prevalence of these impacts on the current situation can improve the population's health and reduce its consequences during COVID-19 and future similar pandemics. Therefore, this is the first study that aims to assess the impact of the extended COVID-19 quarantine on the mental health, especially depressive symptom levels, and the sleep quality of a large sample of undergraduate University students in Jordan. This is assessed by collecting many exposures to cover the demographic, economic, and quarantine-related factors that might worsen the effect of quarantine on both the students' sleep quality and mental health.

## Method

### Participants

The online questionnaire participants were undergraduate students at the University of Jordan (UJ, located in Amman) who voluntarily completed its questions. The total number of collected responses had reached 7,146. Six thousand one hundred fifty-seven unique participants remained after cleaning the data by removing all the duplicated submissions. All the questions were obligatory; hence there was no missing data. At any time, any participant could have ignored answering any of the questions to withdraw from the study. The Institutional Review Board / the Research Ethics Committee at UJ had approved the study objectives and procedures. The age of the participants ranged between 17 and 30, with a mean of 19.79 ± 1.67. Nearly half of the students were in their first year. 28.7% of participants were males (1,769), and 71.3% were females (4,388), with a male to female ratio of 1:2.48. Half of the students were studying humanities-related majors, and 36.2% were studying scientific majors, while 13.6% were from the medical schools (medicine, dentistry, nursing, pharmacy, and rehabilitation sciences).

### Measurements of Clinical Symptoms

The questionnaire collects an extensive list of general socio-demographic, socio-economic, and quarantine-related information (as a measure of exposures) in addition to the questions in the PSQI and CES-D measures to assess the primary outcomes: sleep quality and depressive symptom levels. The reason for collecting this extensive list of exposures was to cover the main confounding factors and assess how these many different exposures may affect/associate with the two primary outcomes.

### Socio-Economic and Socio-Demographic Factors

Socio-economic factors regarding the household income, parents' education levels ranging from “did not reach high school” to “postgraduate,” and parents' employment status during the quarantine were collected. Furthermore, gender, age, year level, academic major/performance, and students' smoking practices were measured.

### Quarantine Variables

To assess the effect of home quarantine on student's mental health, more information related to the stay at home period, including the number of members (and children) quarantined with each student, place of quarantine (rural or urban), house specifications (apartment/independent house with/without a garden), household income during the quarantine, communication with family members, and practiced hobbies were gathered.

### Clinical Assessment of Sleep Quality

The sleep quality of the undergraduate University students during the several weeks of COVID-19 home quarantine was assessed using Pittsburgh's Sleep Quality Index (PSQI) (46). This index is a validated self-reported questionnaire that measures the quality of sleep subjectively from different perspectives. It contains 19 items grouped into seven components, each measures one aspect (Table 1). The components are subjective sleep quality (very good, fairly good, fairly bad, and very bad), sleep latency (time between lying down in bed and falling asleep), duration (<5 h, 5–6 h, 6–7 h, >7 h), efficiency (<65%, 65–74%, 75–84%, >85%), disturbance, the need to use sleep medication (yes, no), and daytime dysfunction. Each component is scored on a four-point scale from 0 (no difficulty) to 3 (severe difficulty). The global score is calculated by adding each component's score and can range from 0 to 21, with higher scores indicating lower sleep quality (46).

**Table 1:** Items of Pittsburgh Sleep Quality Index (PSQI).

	**Component**	**Description**
1	Sleep quality	Perceived overall sleep quality
2	Sleep latency	Measures how long it took to fall asleep
3	Sleep duration	The actual length of sleep
4	Sleep efficiency	The total number of hours slept divided by and the number of hours spent in bed
5	Sleep disturbances	Behaviors that negatively affect sleep, such as waking up at late night or early in the morning, getting up at night to use the bathroom, uncomfortable breathing, coughing or snoring loudly, feeling too hot or too cold, having nightmares, or pain
6	Sleep medication	Whether there is a need to use them to go to sleep
7	Daytime dysfunction	Troubles staying awake while driving, eating meals or engaging in social activity, or keep enough enthusiasm to get thing done

*This scale was proposed by Buysse et al. (46)*.

### Clinical Assessment of Depressive Symptoms

Depressive symptoms were assessed using the Center for Epidemiologic Studies-Depression Scale (CES-D) (47). It is a validated self-reporting scale that contains 20 items, each ranged between 0 and 3 (Table 2). The global score is calculated by adding all items' scores, which ranged from 0 to 60. The four-point scale is: rarely or less than once a day (scores 0 points), some of the time or 1-2 days (scores one point), occasionally or moderate amount of time or 3-4 days (scores two points), and most of the time or 5–7 days (scores three points). The higher the global score is, the higher levels of depressive symptoms there are (47).

**Table 2:** Items of Center for Epidemiologic Studies-Depression Scale (CES-D).

**Items**		**Items**	
1	I was bothered by things that usually don't bother me	11	My sleep was restless
2	I did not feel like eating; my appetite was poor	12	I was happy
3	I felt that I could not shake off the blues even with help from my family or friends	13	I talked less than usual
4	I felt I was just as good as other people	14	I felt lonely
5	I had trouble keeping my mind on what I was doing	15	People were unfriendly
6	I felt depressed	16	I enjoyed life
7	I felt that everything I did was an effort	17	I had crying spells
8	I felt hopeful about the future	18	I felt sad
9	I thought my life had been a failure	19	I felt that people dislike me
10	I felt fearful	20	I could not get “going”

*This scale was proposed by Radloff et al. (47)*.

### Statistical Analysis

Frequencies and percentages were used to analyze the categorical demographic, economic, and quarantine variables, while mean and standard deviation were used for continuous variables. A two-sample *t*-test was used to test for significance for the binary variables, while multi-values variables were tested using a one-way analysis of variance (ANOVA). As a *post-hoc* analysis, Tukey Honestly Significance Difference (TukeyHSD) was used to follow up on the significant factors that resulted from the ANOVA to identify the pair of values that had a significant mean difference. The significant factors were further investigated using logistic regression, and the significant associations between the exposures and the primary outcomes were identified using the Backward selection method. While binary logistic regression was used for the sleep quality state (1: poor, 0: normal), the multinomial logistic regression was used for the depressive symptoms state (1: low, 2: moderate, and 3: high). A *p*-value of ≤ 0.05 was considered to be statistically significant. All statistical analyses were performed using R version 4.0.0 and RStudio version 1.2.5042.

## Results

### Demographic and Economic Characteristics of the Study Participants

Nearly half of the participants (*n* = 3,003) were fresh students, with most (*n* = 3,092) studying humanities-related majors. Around three-quarters were females (*n* = 4,388), and only 16.3% were smokers (*n* = 1,006). The average mean age was 19.79, and the standard deviation was 1.67. Only 3.5% of the students are about to graduate (*n* = 217) (Table 3; the first two columns).

**Table 3:** Socio-demographic and socio-economic characteristics, PSQI and CES-D scores of study participants.

**Variable**	**Mean ± SD or *N* (N%)**	**PSQI ScoreMean ± SD or(*p*-value)**	**CES-D Score Mean ± SD or (*p*-value)**
Age	19.79 ± 1.67	8.1 ± 3.6	22.2 ± 11.7
Gender		(8.34e-04^a^^*^)	(4.02e-07^a^^*^)
Male	1,769 (28.7%)	7.9 ± 3.7	21.0 ± 11.7
Female	4,388 (71.3%)	8.2 ± 3.5	22.6 ± 11.7
Major		(6.28e-06^b^^*^)	(0.941^b^)
Humanities	3,092 (50.2%)	8.4 ± 3.6	22.1 ± 11.9
Medical	840 (13.6%)	7.9 ± 3.6	22.3 ± 11.7
Scientific	2,235 (36.2%)	7.9 ± 3.5	22.2 ± 11.6
Class		(2.69e-05^b^^*^)	(0.472^b^)
Year 1	3,003 (48.8%)	7.9 ± 3.5	21.9 ± 11.7
Year 2	1,757 (28.5%)	8.2 ± 3.6	22.6 ± 11.5
Year 3	793 (12.9%)	8.4 ± 3.7	21.9 ± 12.3
Year 4	481 (7.8%)	8.5 ± 3.7	22.3 ± 11.5
> Year 4 (Year 5, Year 6, and more)	123 (2.0%)	9.1 ± 4.0	22.0 ± 12.4
About to graduate		(8.09e-04^a^^*^)	(0.168^a^)
Yes	217 (3.5%)	9.0 ± 3.8	23.3 ± 12.0
No	5,940 (96.5%)	8.1 ± 3.6	22.1 ± 11.7
Smoking		(3.85-03^a^^*^)	(0.285^a^)
Yes	1,006 (16.3%)	8.4 ± 3.6	22.5 ± 11.9
No	5,151 (83.7%)	8.1 ± 3.6	22.1 ± 11.7
Household Income Level(1 JD = ~1.4 USD)		(8.30e-13^b^^*^)	(0.181^b^)
Less than 200 JD	375 (6.2%)	9.1 ± 3.7	22.7 ± 13.9
200–400 JD	1,225 (19.9%)	8.5 ± 3.6	22.5 ± 12.1
400–600 JD	1,207 (19.6%)	8.2 ± 3.6	22.4 ± 11.6
600–800 JD	951 (15.4%)	8.1 ±3.4	22.2 ± 11.7
800–1,000 JD	955 (15.5%)	7.9 ± 3.4	22.2 ± 11.3
1,000–1,200 JD	493 (8.0%)	7.8 ± 3.5	22.2 ± 11.2
1,200–1,500 JD	341 (5.5%)	7.5 ± 3.6	20.9 ± 11.1
More than 1,500 JD	610 (9.9%)	7.7 ± 3.8	21.2 ± 11.2
Education level (Father)		(0.011^b^^*^)	(0.031^b^^*^)
Post graduates	732 (11.9%)	8.0 ± 3.6	21.5 ± 11.8
Bachelor	2,066 (33.6%)	8.0 ± 3.6	21.7 ± 11.4
Diploma	1,126 (18.3%)	8.2 ± 3.5	22.8 ± 11.6
High School	1,485 (24.1%)	8.3 ± 3.5	22.5 ± 12.0
Others (did not reach high school)	748 (12.1)	8.4 ± 3.6	22.4 ± 12.1
Education level (Mother)		(6.72e-03^b^^*^)	(0.502^b^)
Post graduates	308 (5.0%)	7.9 ± 3.8	21.3 ± 12.0
Bachelor	1,779 (28.8%)	8.0 ± 3.7	22.0 ± 11.5
Diploma	1,543 (25.1%)	8.2 ± 3.5	22.4 ± 11.9
High school	1,900 (30.9%)	8.2 ± 3.5	22.1 ± 11.7
others (did not reach high school)	627 (10.2%)	8.5 ± 3.7	22.6 ± 12.2

*Total number of participants: 6,157 students,—SD, Standard Deviation*.

*Numerical variables were summarized as mean and standard deviation, while the categorical variables were summarized using percentages*.

*PSOI, Pittsburgh's Sleep Quality Index (46)*.

*CES-D, Center for Epidemiologic Studies Depression Scale (47)*.

a p-value is obtained using t-test;

b *p-value is obtained using one-way-ANOVA*.

* *Statistically significant p-value (≤ 0.05)*.

Furthermore, around 45% (*n* = 2,798) and 34% (*n* = 2,087) of the students' fathers and mothers had a University degree (bachelor or postgraduate). The household income level ranged from <200 JD ($ 282) to more than 1,500 JD ($ 2,115), which mainly fall into three categories; very low to low income (<600 JD: 45%, *n* = 2,807), medium income (600–1,000 JD: 30%, *n* = 1,906), and high income (more than a 1,000 JD: 25%, *n* = 1,444) (Table 3; the first two columns).

### Quarantine Characteristics of the Study Participants

Only 4.5% (*n* = 275) of the students had their household income increased during the quarantine, whereas nearly 50% had either a decreased or a completely stopped income (*n* = 2,467 and *n* = 775, respectively). A low proportion of 13.7% (*n* = 842) of the students were quarantined in rural areas. 55% (*n* = 3,350) lived in an apartment; one-third of these apartments had a garden. The majority (~80%, *n* = 2,210) of the students who lived in an independent house had a garden (Table 4; the first two columns). Watching movies and/or TV series in addition to sleeping were the most common activities (70%, *n* = 4,310) among the students during the quarantine, and then eating or cooking with a percentage of nearly 50% (*n* = 3,079). More than half of the students (68%, *n* = 4,187) start practicing new hobbies like board games (25%, *n* = 1,539), drawing (11%, *n* = 677), cooking (42%, *n* = 2,586), meditation (16%, *n* = 985) and watching movies/series (51%, *n* = 3,140). Despite the different demographics for the students, the majority of them (89.7%, *n* = 5,523) communicated more with their families and reported that they are spending more time with their families during the quarantine, and around 70% (*n* = 4,310) increased their communication with the members living apart. Furthermore, students were asked about the health of the family members and friends that they were quarantined with; more than half of the students reported that they were quarantined with a smoker (*n* = 3,386), around 20% (*n* = 1,416) with a diabetic patient, about 8% (*n* = 493) with a cardiac patient, and 17% (*n* = 1,047) with an elderly member (>65 years). Finally, during the quarantine, 77% (*n* = 4,741) of the students lived with 3–7 family members, and 43% (*n* = 2,648) were not quarantined with children.

**Table 4:** Study participants statistics of quarantine factors, and their corresponding PSQI and CES-D scores.

**Variable**	***N* (N%)**	**PSQI Score Mean ± SD**	**CES-D Score Mean ± SD**
Location of house during quarantine		(0.205^a^)	(0.806^a^)
Urban areas	5,315 (86.3%)	8.1 ± 3.5	22.2 ± 11.6
Rural areas	842 (13.7%)	8.3 ± 3.7	22.1 ± 12.4
Home specification		(1.36e-07^b^^*^)	(0.572 ^b^)
Apartment with garden	1,176 (19.1%)	8.0 ± 3.5	22.0 ± 11.3
Apartment without a garden	2,174 (35.3%)	8.0 ± 3.5	22.4 ± 11.6
House with garden	2,210 (35.9%)	8.1 ± 3.6	22.0 ± 11.9
House without a garden	597 (9.7%)	9.0 ± 3.7	22.2 ± 12.3
Household income during quarantine		(4.37e-10^b^^*^)	(2.21e-07^b^^*^)
Increased	275 (4.5%)	8.6 ± 3.9	21.2 ± 11.2
Stay the same	2,640 (42.9%)	7.8 ± 3.5	21.2 ± 11.4
Decreased	2,467 (40.1%)	8.3 ± 3.5	23.0 ± 11.8
Stopped completely	775 (12.5%)	8.6 ± 3.7	23.0 ± 12.3
Number of people quarantined with		(1.49e-03^b^^*^)	(0.093^b^)
Less than 4	941 (15.3%)	8.1 ± 3.7	21.6 ± 11.2
4–7	4,248 (69.0%)	8.1 ± 3.5	22.1 ± 11.7
8–10	845 (13.7%)	8.5 ± 3.6	22.7 ± 12.4
More than 10	123 (2.0%)	8.7 ± 3.8	23.8 ± 13.4
Number of children quarantined with		(5.58e-15^b^^*^)	(2.26e-06^b^^*^)
None	2,624 (42.6%)	7.9 ± 3.6	21.5 ± 11.4
1	1,447 (23.5%)	8.1 ± 3.4	22.0 ± 11.1
2	1,120 (18.2%)	8.3 ± 3.5	22.3 ± 12.2
3	513 (8.3%)	8.3 ± 3.5	23.9 ± 12.0
4–6	395 (6.4%)	9.1 ± 4.1	24.4 ± 12.9
More than 6	58 (1.0%)	10.8 ± 4.4	22.6 ± 16.7

*Total number of participants: 6,157 students*.

*SD, Standard Deviation*.

*Numerical variables were summarized as mean and standard deviation, while the categorical variables were summarized using percentages*.

*PSOI, Pittsburgh's Sleep Quality Index (46)*.

*CES-D, Center for Epidemiologic Studies Depression Scale (47)*.

a *p-value is obtained using t-test*.

b *p-value is obtained using one-way-ANOVA*.

* *Statistically significant p-value (≤ 0.05)*.

### Psychological Findings of the Study Participants (Sleep Quality)

Students' sleeping behaviors were assessed through the PSQI. It revealed an evident abnormal and unhealthy sleeping habits, which might affect sleep quality. For instance, more than three-quarters of the students (77%, *n* = 4,764) went to bed after midnight during the quarantine, more than half of them (*n* = 2,711) went to bed after 3 a.m. About half of the students (*n* = 3,003) needed more than 30 min to fall asleep after going to bed, and 30% (*n* = 1,847) needed more than 40 min. Sixty percentage of the students (*n* = 3,669) woke up after midday and 33% (*n* = 2,031) woke up after 2 p.m. Forty percentage of the students (*n* = 2,463) slept for more than 9 h and around 8% (*n* = 493) slept more than 12 h a day.

More than one-fifth (*n* = 1,416) of the students had to take medications to help them sleep during the quarantine. Around half of the students (*n* = 3,196) experienced difficulties staying awake while doing a daytime activity. Furthermore, around 80% (*n* = 4,870) of the students found it challenging to stay enthusiastic in order to complete tasks during the quarantine (30%; *n* = 1,866, reported that this had been a minor problem, another 30%; *n* = 1,865, found this somewhat of a problem, and about 20%; *n* = 1,139, stated that this was a big problem they suffer from). According to self-reporting, nearly half of the students (*n* = 3,060) had poor sleep quality (12.1%; *n* = 745 very good, 38.2%; *n* = 2,352 good, 27%; *n* = 1,662 bad, and 22.7%; *n* = 1,398 very bad).

**Figure 1:**
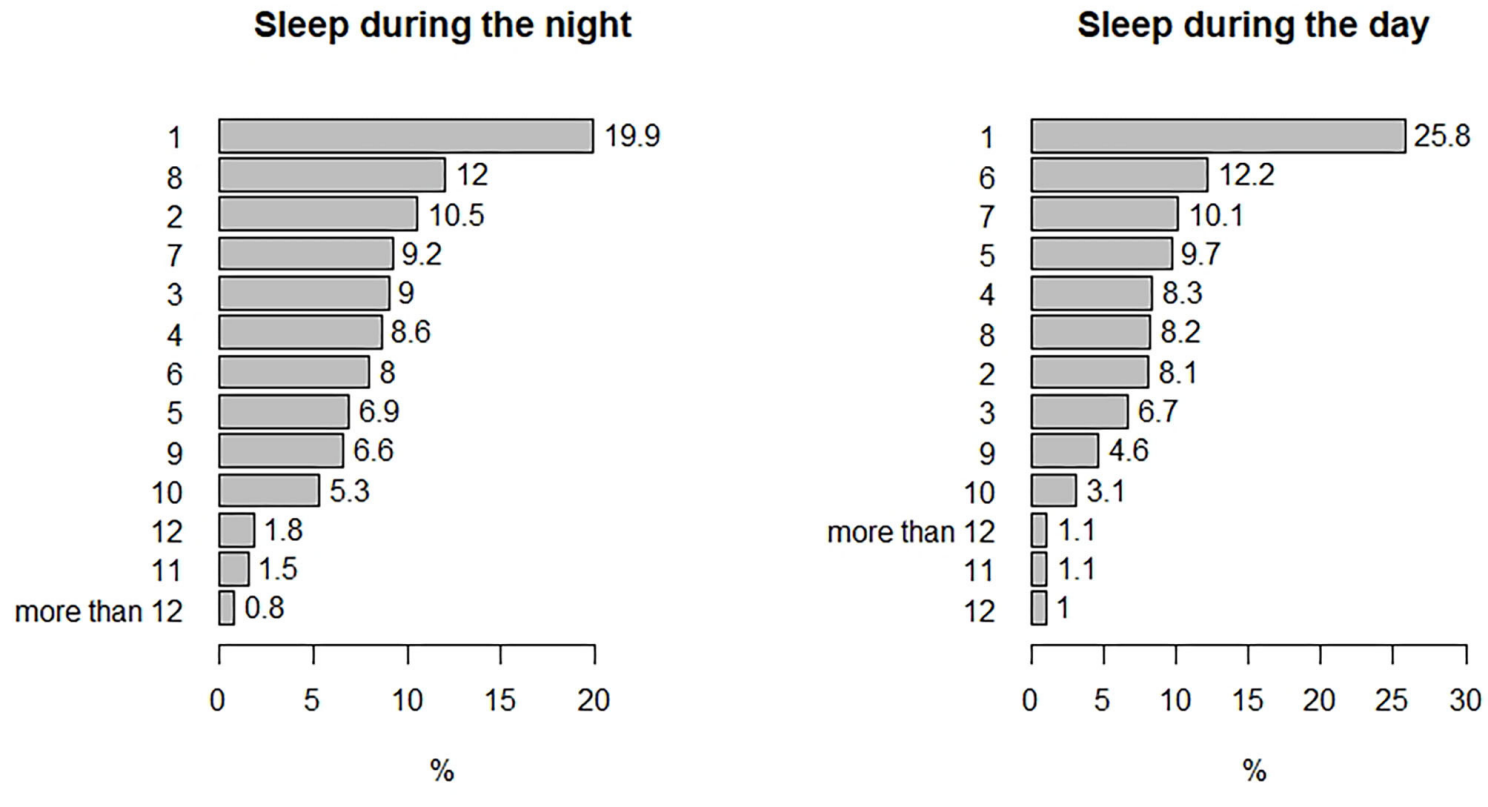
Percentages of the number of sleeping hours reported by the students during the nights and during the days of the quarantine.

Other than the PSQI 19 items, the students were asked a few more questions regarding their sleeping habits during the quarantine. Almost all students (94.9%, *n* = 5,843) reported that the quarantine affected their sleeping times (greatly: 72.5%; *n* = 4,464, slightly: 22.5%; *n* = 1,380), only 5.1% (*n* = 316) were not affected. Around 65% (*n* = 4,002) reverse their sleeping habits as they used to sleep most of the day and woke up most of the night during the quarantine. About 40% (*n* = 2,421) of the students slept 3 h or less, around 10% (*n* = 584) slept more than 10 h, and around 30% (*n* = 1,803) slept more than 7 h during the day (Figure 1). Finally, only 10% (*n* = 611) reported that quarantine affected their sleeping habits positively, whereas 74% (*n* = 4,539) were negatively affected, while the rest (*n* = 1,010) were not affected.

The PSQI mean scores for the different socio-demographic, socio-economic, and quarantine variables are presented in Tables 3, 4, with an overall mean score of 8.1 ± 3.6. The lowest PSQI score was 7.5 ± 3.6 reported by the students with a household income level of 1,200–1,500 JD (Table 3), while the highest score was 10.8 ± 4.4 reported by the students who were quarantined with more than six children (Table 4). A global PSQI score higher than 5 points indicates poor sleep quality (46). Thus, the prevalence of poor sleep quality among participants was 76% (*n* = 4,680), with a mean PSQI score of 9.5 and a standard deviation of 2.9. The prevalence of poor sleep quality in male students was 71.5% (*n* = 1,264) and in females was 77.8% (*n* = 3,416) with very close PSQI scores of 9.6 ± 3.0 and 9.5 ± 2.9 for males and females, respectively (Table 5).

**Table 5:** Sleep quality and depressive symptoms prevalence among the study participants based on PSQI and CES-D scores, respectively.

**Participant groups**	**Factor**	**Prevalence as *N* (N%)**	**PSQI or CES-D Score as mean ± SD**
Poor sleep quality	Male	1,264 (71.5%)	9.6 ± 3.0
	Female	3,416 (77.8%)	9.5 ± 2.9
	Total	4,680 (76.0%)	9.5 ± 2.9
Good sleep quality	Male	505 (28.5%)	3.7 ± 1.3
	Female	972 (22.2%)	3.8 ± 1.1
	Total	1,477 (24.0%)	3.8 ±1.2
Low depressive symptoms (CES-D score <16)	Male	569 (32.2%)	8.9 ± 5.3
	Female	1,201 (27.3%)	9.5 ± 5.2
	Total	1,770 (28.7%)	9.3 ± 5.2
Moderate depressive symptoms (16 ≤ CES-D score ≤ 24)	Male	597 (33.7%)	19.6 ± 2.5
	Female	1,503 (34.3%)	19.9 ± 2.4
	Total	2,100 (34.1%)	19.8 ± 2.5
High depressive symptoms (CES-D score > 24)	Male	603 (34.1%)	33.7 ± 8.1
	Female	1,684 (38.4%)	34.5 ± 7.9
	Total	2,287 (37.2%)	34.3 ± 8.0

*Total number of participants: 6,157 students: 1,769 males, and 4,388 females*.

*SD, Standard Deviation*.

*PSOI, Pittsburgh's Sleep Quality Index (46)*.

*Sleep quality cut-off value (poor quality: PSQI score > 5) is based on what was reported in Buysse et al. (46)*.

*CES-D, Center for Epidemiologic Studies Depression Scale in Radloff (47)*.

*Participant groups division is based on what was reported in Liu et al. (45)*.

The only non-significant binary exposure was the quarantine's house location (*t*-test *p*-value: 0.2: Table 4). Other binary exposures, like gender, graduation status, and smoking habit, were significant (*t*-test *p*-value < 0.05). Females, students in their final University semester, and smokers had a significant association with poor sleep quality than their inverse (Table 3). Students' field of study was also significantly associated with poor sleep quality (ANOVA *p*-value: 6.28e-06), where the mean difference between the humanities and each of the scientific and medical majors were significant (TukeyHSD *p*-values: 2.9e-05 and 2.5e-3, respectively). The humanities-related majors had a larger PSQI mean score than the scientific and medical majors (Table 3).

Besides, students' year of study was significantly associated with poor sleep quality (ANOVA *p*-value: 2.69e-05: Table 3), with the most significant difference between fresh students and those in their third, fourth, and fifth years. The economic status was significantly negatively associated with poor sleep quality (ANOVA *p*-value: 8.30e-13: Table 3), where the most significant mean difference was between lower and higher incomes. Similarly, the parents' education level was inversely associated with the PSQI scores (Table 3), with the significant difference between University degrees and school degrees. Furthermore, house specifications were found significantly associated with sleep quality (ANOVA *p*-value: 1.36e-07: Table 4); the highest PSQI scores were for those living in a house without a garden (9.0 ± 3.7). The income status during the quarantine had a significant association with the PSQI (ANOVA *p*-value: 4.37e-10: Table 4); when income stayed the same, the PSQI was the lowest (7.8 ± 3.5). Finally, the number of people and children quarantined with the student affected the poor sleep quality directly, such that the larger the number of the quarantined members, the higher the PSQI scores and thus the lower sleep quality (Table 4).

All significant exposures (resulting from the pair-wise *t*-test/ANOVA) were combined into one model and analyzed using logistic regression (Table 6) to assess each factor's association with the poor sleep quality after controlling other factors. Females, students in their final semester, smokers, lower household income, living in a house without a garden, decreased income during the quarantine, and being quarantined with more than four children all have a significant association and a potentially higher risk of suffering from poor sleep quality (Table 6). The model was evaluated using the Backward selection method with an Akaike Information Criterion (AIC) of 6692.8 and a difference of 127.6 between residual and null deviance with 17 degrees of freedom.

**Table 6:** Association between poor sleep quality state and each of the identified significant exposures, as assessed by logistic regression^+^.

**Coefficients**	**Estimate**	***p*-value**	**Odd ratio**	**CI lower**	**CI upper**
(Intercept)	0.827	5.41e−15^*^	2.287	0.621	1.036
Sex (Male)	−0.426	2.22e−09^*^	0.653	−0.565	−0.286
Graduation semester (yes)	0.404	0.027^*^	1.498	0.057	0.776
Smoking (yes)	0.360	8.01e−05^*^	1.434	0.183	0.541
Household income (0–200 JD)	0.726	2.74e−05^*^	2.068	0.392	1.072
Household income (200–400 JD)	0.371	0.002^*^	1.449	0.141	0.600
Household income (400–600 JD)	0.267	0.019^*^	1.307	0.043	0.491
Household income (600–800 JD)	0.237	0.045^*^	1.268	0.004	0.469
Household income (800–1,000 JD)	0.299	0.012^*^	1.348	0.066	0.530
Household income (1,000–1,200 JD)	0.134	0.328	1.143	−0.133	0.402
Household income (1,200–1,500 JD)	−0.180	0.218	0.835	−0.467	0.108
Home specification (Apart. without a garden)	−0.054	0.449	0.947	−0.194	0.086
Home specification (Apart. with a garden)	−0.001	0.989	0.999	−0.168	0.167
Home specification (House without a garden)	0.325	0.007^*^	1.383	0.092	0.564
Income during quarantine (Stopped)	0.097	0.341	1.101	−0.101	0.297
Income during quarantine (Increased)	0.175	0.253	1.191	−0.118	0.481
Income during quarantine (Decreased)	0.255	0.0001^*^	1.290	0.124	0.386
Quarantine with more than four children	0.516	0.009^*^	1.675	0.145	0.917

*CI: Confidence Interval*.

* *Statistically significant p-value (≤ 0.05)*.

+ *Dependent variable: poor sleep quality state; calculated based on the suggested PSQI scores threshold of > 5, reported in Buysse et al. (46)*.

*Baseline for Household Income is “more than 1,500 JD”*.

*Baseline for Home specification is “House with a garden”*.

Baseline for Income during quarantine is “Stayed the same”

### Psychological Findings of the Study Participants (Depressive Symptoms)

The CES-D mean scores for the different socio-demographic, socio-economic, and quarantine variables are presented in Tables 3, 4, with an overall mean score of 22.2 ± 11.7. However, students were divided into three groups based on their CES-D scores as suggested by a study on depression levels for hospital employees after the 2003 SARS epidemic (45); low level of depressive symptoms group with a CES-D score of <16, moderate level of depressive symptoms group with CES-D score between 16 and 24, and high level of depressive symptoms group with a CES-D score of >24. The prevalence of moderate and high depressive symptoms was higher in female students (34.3 and 38.4%, respectively) than the male students. Similarly, the CES-D mean scores were higher in females in all groups than their male colleagues (Table 5). The overall mean CES-D score for the low depressive symptoms group is 9.3, for the moderate group is 19.8, while it is 34.3 for the high symptoms group (Table 5).

More than half of the students (62.5%, *n* = 3,851) reported that the quarantine had a negative effect on their mental health, and only 10.4% (*n* = 640) reported the opposite, whereas the rest (27.1%, *n* = 1,666) were not affected. Around one-fifth (*n* = 1,285) of the students reported a change in their attitude by becoming more anxious with hard-tempered than they used to be, while about one-tenth (*n* = 596) reported a change in the opposite direction.

Using pair-wise *t*-test/ANOVA, only four factors were significantly associated with high depressive symptom levels; the gender (*t*-test *p*-value: 4.02e-07: Table 3), father's education level (ANOVA *p*-value: 0.031: Table 3), household income during quarantine, and number of children quarantined with (ANOVA *p*-values: 2.21e-07 and 2.26e-06, respectively: Table 4). However, the multinomial logistic regression results used to control for confounding factors and study the combined effect of the different exposures on the depressive symptoms state show a different pattern. Female students are more likely to suffer from moderate (Wald test *p*-value: 6.08e-03) and high (Wald test *p*-value: 4.55e-07) depressive symptoms than male students. Furthermore, smokers and students with decreased income during quarantine have higher risks for developing high depressive symptoms than their counterparts with Wald test *p*-values of 7.78e-04 and 5.58e-07, respectively.

## Discussion

This study's participants were students from the University of Jordan, the largest public University in Jordan, Amman. UJ hosts about 35,000 students studying undergraduate and postgraduate degrees in humanities, science, and health disciplines. Seventy-six percent of the UJ students are females, and about half of the students (50.3%) study humanities-related majors. The total number of participants in this study was 6,157 students (represent 18% of the whole University students) who filled the online questionnaire. The questionnaire link was uploaded as part of several University compulsory courses which are mainly covered during the first 2 years of the majors, thus, explaining why around 77% of the participants were in year 1 and year 2, with a mean age of 20 years, whereas only 3.5% of the students were in their final semester (Table 3). This sample of participants is a good representative of the University demographics as 71.3% of the study participants are females, and 50.2% are studying humanities.

Moreover, this sample is representative of the Jordanian population. According to the national survey conducted by the National Council for Family Affairs (NCFA) in 2017 (48), about 78% of the families that participated had 3–7 members, which is comparable to sample study demographics (Table 4). Furthermore, according to the NCFA survey, about 57 and 42% of the families that participated lived in apartments and separate houses. This is consistent with the current study in which the students reported percentages of 54.4 and 45.6% correspondingly (Table 4). Regarding chronic diseases, non-communicable chronic diseases (NCCD) prevail in the society, as 14.5 and 7.2% suffer from diabetes and cardiovascular diseases, respectively. In this sample, 23 and 8% of the students were quarantined with a family member suffering from diabetes and cardiovascular diseases, respectively. Additionally, as reported by WHO (49), tobacco smoking is more prevalent in Jordanian males, where 70% of males aged more than 14 years are smokers (50). This explains the high percentage of nearly half of the students who were quarantined with a smoker. The preponderance of females who participated might account for the 16.3% reported smoker status (Table 3). Nevertheless, around 70% of the student participants were females. Although this represents the UJ community (public universities tend to admit students with high grades, which is more achievable by females than males in Jordan), it is not representative of the University student population in Jordan. This potential selection bias was controlled by logistic regression.

The impact of the extended quarantine on students' sleeping behavior is tremendously apparent. 94.9% of the students reported that their sleeping habits were affected; 74% in a negative way, especially in reversing the day-night activities (65%) and highly increasing or decreasing the quantity of sleeping hours, which resulted in reducing the quality of their sleep (~50%). These results can be explained by the staying-at-home order, distance-learning/working, banning outdoor activities, COVID-19 updates news all over the media, the broad and unprecedented closure, and many more different forced lifestyles, which affected the well-being of most if not all the Jordanians. All these factors contributed to the high prevalence of sleeping disorders among the participants, reaching 76% of the sample. The gender was significantly associated with lower sleep quality (Table 6; logistic regression coefficient *p*-value: 2.33e-09) and had significantly higher PSQI scores (Table 3; *t*-test *p*-value: 8.34e-04), with a clear difference in the prevalence between male (71.5%) and female (77.8%) students, which is aligned with the reported literature (51–54). However, a few studies reported the opposite (55, 56).

Furthermore, this study revealed that smokers had significantly lower sleep quality than non-smokers (Table 6; logistic regression coefficient *p*-value: 8.01e-05). A cross-sectional study from central China's general population reported that smokers demonstrated lower sleep quality and more sleeping disturbances, a finding supported by a plethora of other studies (57–60). One plausible explanation would be tobacco's effect and the changes it induces to the core circadian clock gene expression, which affects sleeping habits (61, 62). Likewise, the significant correlation between lower incomes and poor sleep quality (Table 6) is consistent with previous studies (53, 63, 64).

The parameters related to the University-study variables, including the effect of the study major, and year of study, impacted the sleep quality. The pair-wise significant association between studying in humanities and poor sleep quality when compared to medical and scientific students (Table 3; ANOVA *p*-value: 6.28e-06) as reported in this study contradicts what was reported in an abstract presented in SLEEP 2007; the 21st Annual Meeting of the Associated Professional Sleep Societies (APSS). It revealed that medical students suffer more from poor sleep quality than their peers in humanities majors (65). More-so, the pair-wise significant difference in respect to students' year of study (with the most significant difference between fresh students who had relatively better sleep and those in their third, fourth, and fifth years) is also consistent with a study of 860 medical students from 49 medical colleges in the United States, which revealed higher rates of sleeping disorders in first- and third-year students relative to second- and fourth-year students (66). It is not surprising that students in their final semester, or with low household income or decreased income during the unprecedented closure, significantly suffer from sleeping disturbances more than their peers (Table 6). Likewise, when the number of children the student quarantined with increase, their sleep quality decrease (Tables 4, 6). Interestingly, living in a house without a garden resulted in lower sleep quality (Table 6: logistic regression coefficient *p*-value: 0.009).

The assessment of the depressive symptoms among the Jordanian students is alarming as the prevalence of the high/and potentially-high risk group that showed high/and moderate depressive symptoms was 37.2 and 34.1%, respectively, with a total risk percentage of 71.3%. This is comparable to the 74.3% prevalence reported in Greece (23). Uncertainty and unclear plans for the academic semester and the grades probably left the students anxious and stressed. Besides, social distancing and lack of social communication may have affected the students with loneliness and isolation, ultimately leading to more depressive symptoms and sad feelings. The female gender is considered a significant risk factor for high depressive symptoms (logistic regression coefficient *p*-value: 4.55e-07). The susceptibility of females to develop depressive symptoms was also reported in previous studies (67, 68). Female sensitivity to stress might be explained by the role sex steroids play in mood regulation (69). Depressive symptoms in low-income families were prevalent, regardless of quarantine (70, 71). During the quarantine, the effect of the sudden closure and losing the source of income with a lack of savings can lead to an unstable and stressful financial state. So, decreased income during quarantine is also significantly linked with higher depressive symptoms (logistic regression coefficient *p*-value: 5.58e-07). In addition, two previous studies conducted in Southwestern China and Canada showed similar findings; high levels of anxiety and depressive symptoms were correlated with low average household income (43, 72). Students in their final semester did not show significantly higher depressive symptoms than their colleagues (both categories had high CES-D scores; Table 3), albeit a study of home-quarantined students in China reported the opposite (73).

**Figure 2:**
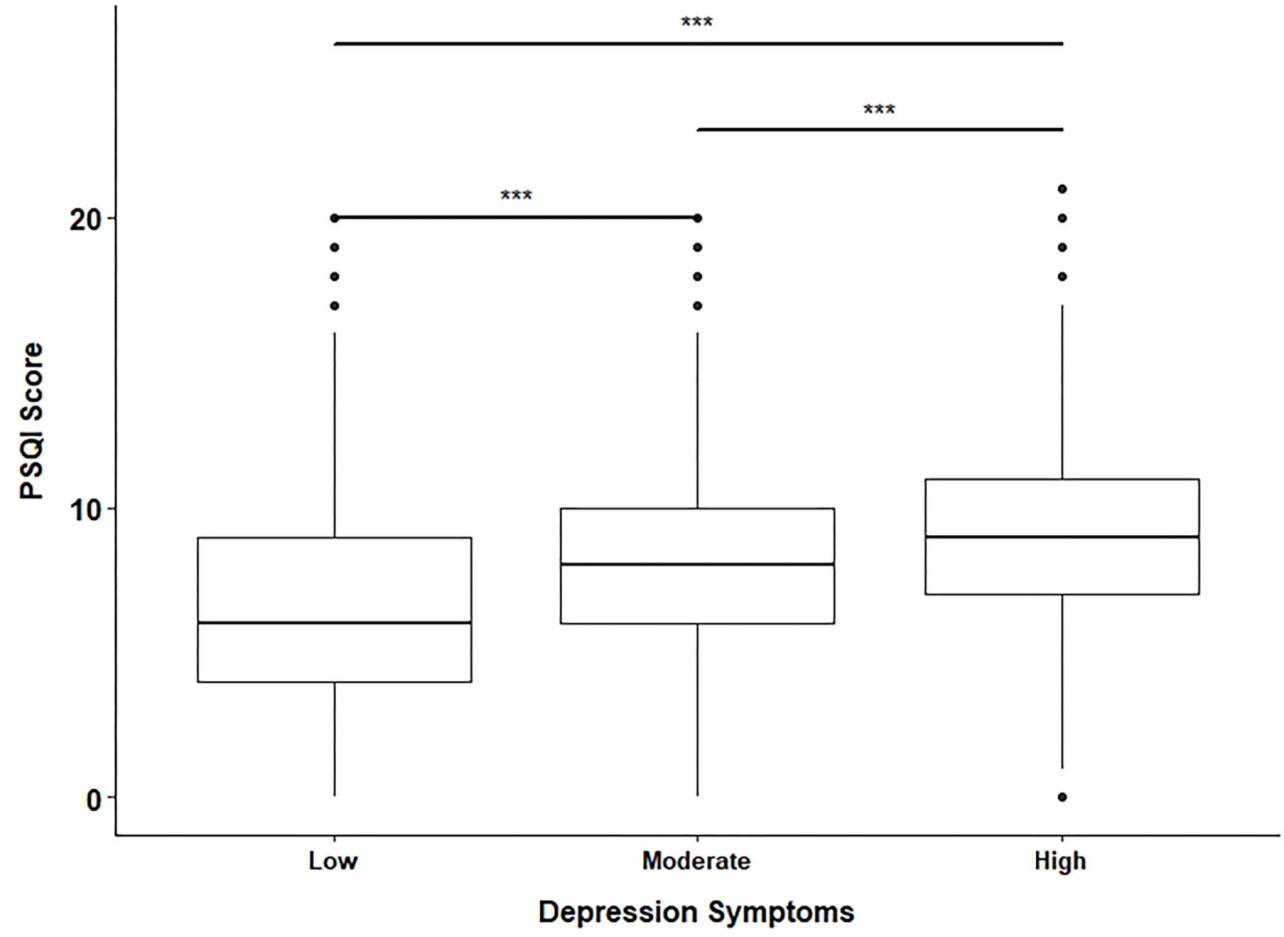
Box plot for PSQI scores of the three groups of depressive symptoms levels. The low depressive symptoms group was determined by a CES-D score <16, the moderate had a CES-D score between 16 and 24, while the high group had a score >24. The pair-wise comparisons between the three groups were significant. The *p*-values from the *t*-test were all < 0.001 (***).

Finally, poor sleep quality is a risk factor for many chronic diseases' incidence and progression and psychological problems, including depression, anxiety, and suicidal behavior (64, 74–80). According to Celik et al. the risk of depressive symptoms in students with poor sleep quality was 3.28 times higher (81). This is consistent with this study's finding, as there was a positive correlation between the PSQI scores and the severity of the depressive symptoms (Figure 2). In addition, non-pharmacological sleep interventions were found to be effective in reducing the severity of clinical depressive symptoms (82). Thus, engagement in healthy life patterns, including exercise, might help tackle these serious issues.

We acknowledge the limitations of this study. The sample was drawn from one University in the capital city of Amman. The quarantine effects, including sleep quality and depressive symptoms, could differ in other cities in Jordan. Also, the preponderance of earlier University year's students could have skewed the results. One significant limitation is the potential selection bias resulted from having around 70% of female participants. More balanced selection criteria would be better to apply. However, this factor was controlled in the logistic regression model. Another significant limitation is related to the deficiency of literature on the sleep quality and depressive symptoms scales before the quarantine on the Jordanian population, thus hindering any comparison outside the quarantine period. We recommend that this study be repeated outside the quarantine period, in other areas outside Amman, and to target older University students. Nevertheless, a recent pre-quarantine study reported moderate depressive symptom levels for 600 University students in Jordan using the Depression, Anxiety, and Stress Scale (DASS-21) (83).

## Conclusion

In conclusion, this is the first study that evaluated the effect of the COVID-19 pandemic and the resultant quarantine among University students in Jordan. Poor sleep quality and depressive symptoms were prevalent among this group of participants. The results of this study should be taken seriously to address and guide policy-makers and authorities when planning for extended closures and lock-down. Repeating the study outside the COVID-19 pandemic might help to quantify these issues among University students better. The COVID-19 pandemic has infringed on many aspects of our lives. This has gone beyond the economic into the mental and psychological reverberation.

## Data Availability Statement

The raw data supporting the conclusions of this article will be made available by the authors, without undue reservation.

## Ethics Statement

The studies involving human participants were reviewed and approved by Institutional Review Board and the Research Ethics Committee at UJ. The ethics committee waived the requirement of written informed consent for participation.

## Author Contributions

HS and MS conceived the idea, performed the analysis, and wrote the manuscript. MA-H co-wrote the manuscript and helped in design the study. WA, AA, NS, RA, HK, and SA-S contributed to collect the data and to the literature search. All authors contributed to the article and approved the submitted version.

## Conflict of Interest

The authors declare that the research was conducted in the absence of any commercial or financial relationships that could be construed as a potential conflict of interest.

## References

[1] Guan W Ni Z Hu Y Liang W Ou C He J. Clinical characteristics of coronavirus disease 2019 in China. N Engl J Med. (2020) 382:1708–20. 10.1056/NEJMoa2002032 32109013 PMC7092819

[2] Coronavirus Disease (COVID-19) Situation Reports [Internet]. Available online at: https://www.who.int/emergencies/diseases/novel-coronavirus-2019/situation-reports (accessed December 20, 2020)

[3] Sabat I Neuman-Böhme S Varghese NE Barros PP Brouwer W van Exel J. United but divided: policy responses and people's perceptions in the EU during the COVID-19 outbreak. Health Policy. (2020) 124:909–18. 10.1016/j.healthpol.2020.06.009 32631613 PMC7307992

[4] Jefferson T Foxlee R Del Mar C Dooley L Ferroni E Hewak B. Interventions for the interruption or reduction of the spread of respiratory viruses. Cochrane Database Syst Rev. (2007) 17:CD006207. 10.1002/14651858.CD006207.pub2 17943895

[5] Dénes A Gumel AB. Modeling the impact of quarantine during an outbreak of Ebola virus disease. Infect Dis Model. (2019) 4:12–27. 10.1016/j.idm.2019.01.003 30828672 PMC6382747

[6] Hou C Chen J Zhou Y Hua L Yuan J He S. The effectiveness of quarantine of Wuhan city against the Corona Virus Disease 2019 (COVID-19): a well-mixed SEIR model analysis. J Med Virol. (2020) 92:841–8. 10.1002/jmv.25827 32243599

[7] Nussbaumer-Streit B Mayr V Dobrescu AI Chapman A Persad E Klerings I. Quarantine alone or in combination with other public health measures to control COVID-19: a rapid review. Cochrane database Syst Rev. (2020) 4:CD013574. 10.1002/14651858.CD013574.pub2 32267544 PMC7141753

[8] Tang B Xia F Tang S Bragazzi NL Li Q Sun X. The effectiveness of quarantine and isolation determine the trend of the COVID-19 epidemics in the final phase of the current outbreak in China. Int J Infect Dis. (2020) 95:288–93. 10.1016/j.ijid.2020.03.018 32171948 PMC7162790

[9] Efficiency of quarantine during an epidemic of severe acute respiratory syndrome Beijing, China, 2003 Vol. 52, Morbidity and Mortality Weekly Report. (2003). p. 1037–40. 14586295

[10] Sahu P. Closure of universities due to coronavirus disease 2019 (COVID-19): impact on education and mental health of students and academic staff. Cureus. (2020) 12:e7541. 10.7759/cureus.7541 32377489 PMC7198094

[11] Brooks SK Webster RK Smith LE Woodland L Wessely S Greenberg N. The psychological impact of quarantine and how to reduce it: rapid review of the evidence. Lancet. (2020) 395:912–20. 10.1016/S0140-6736(20)30460-8 32112714 PMC7158942

[12] Mattioli AV Ballerini Puviani M Nasi M Farinetti A. COVID-19 pandemic: the effects of quarantine on cardiovascular risk. Eur J Clin Nutr. (2020) 74:852–5. 10.1038/s41430-020-0646-z 32371988 PMC7199203

[13] Nicola M Alsafi Z Sohrabi C Kerwan A Al-Jabir A Iosifidis C. The socio-economic implications of the coronavirus pandemic (COVID-19): a review. Int J Surg. (2020) 78:185–93. 10.1016/j.ijsu.2020.04.018 32305533 PMC7162753

[14] Gilat R Cole BJ. COVID-19, medicine, and sports. Arthrosc Sports Med Rehabil. (2020) 2:e175–6. 10.1016/j.asmr.2020.04.003 PMC7151370 32292914

[15] Alhola P Polo-Kantola P. Sleep deprivation: impact on cognitive performance. Neuropsychiatric Dis Treatment. (2007) 3:553–67. 19300585 PMC2656292

[16] Chattu V Manzar M Kumary S Burman D Spence D Pandi-Perumal S. The global problem of insufficient sleep and its serious public health implications. Healthcare. (2018) 7:1. 10.3390/healthcare7010001 30577441 PMC6473877

[17] Chaput J-P Wong SL Michaud I. Duration and quality of sleep among Canadians aged 18 to 79. Heal Rep. (2017) 28:28–33. 28930365

[18] Berhanu H Mossie A Tadesse S Geleta D. Prevalence and associated factors of sleep quality among adults in jimma town, southwest ethiopia: a community-based cross-sectional study. Sleep Disord. (2018) 2018:8342328. 10.1155/2018/8342328 29850261 PMC5937373

[19] Olawale O Taiwo O Hesham A. Quality of sleep and well-being of health workers in Najran, Saudi Arabia. Indian J Psychiatry. (2017) 59:347–51. 10.4103/psychiatry.IndianJPsychiatry_241_16 29085095 PMC5659086

[20] Hartley S Colas des Francs C Aussert F Martinot C Dagneaux S Londe V. The effects of quarantine for SARS-CoV-2 on sleep: an online survey. Encephale. (2020) 46:S53–9. 10.1016/j.encep.2020.05.003 32475692 PMC7211567

[21] Casagrande M Favieri F Tambelli R Forte G Authors C. Journal Pre-proof The enemy who sealed the world: effects quarantine due to the COVID-19 on sleep quality, anxiety, and psychological distress in the Italian population. The enemy who sealed the world: Effects quarantine due to the COVID-19 on sleep quality, anxiety, and psychological distress in the Italian population. Background. (2020) 25:698–701. 10.2139/ssrn.357680532853913

[22] Yuan S Liao Z Huang H Jiang B Zhang X Wang Y. Comparison of the indicators of psychological stress in the population of hubei province and non-endemic provinces in China during two weeks during the coronavirus disease 2019 (COVID-19) outbreak in february (2020). Med Sci Monit. (2020) 26:1–10. 10.12659/MSM.923767 32294078 PMC7177041

[23] Kaparounaki CK Patsali ME Mousa D-P V Papadopoulou EVK Papadopoulou KKK Fountoulakis KN. University students' mental health amidst the COVID-19 quarantine in Greece. Psychiatry Res. (2020) 290:113111. 10.1016/j.psychres.2020.113111 32450416 PMC7236729

[24] Suso-Ribera C Martín-Brufau R. How Much Support Is There for the Recommendations Made to the General Population during Confinement? A Study during the First Three Days of the COVID-19 Quarantine in Spain. 32570832 10.3390/ijerph17124382 PMC7345636

[25] World Health Organization. Depression [Internet]. (2020). Available onlin at: https://www.who.int/news-room/fact-sheets/detail/depression (accessed August 21, 2020).

[26] Twenge JM Cooper AB Joiner TE Duffy ME Binau SG. Age, period, and cohort trends in mood disorder indicators and suicide-related outcomes in a nationally representative dataset, 2005-2017. Vol. 128. J Abnormal Psychol. (2019) 128:185–99. 10.1037/abn0000410 30869927

[27] Twenge JM Joiner TE Rogers ML Martin GN. Increases in depressive symptoms, suicide-related outcomes, and suicide rates among U.S. Adolescents after 2010 and links to increased new media screen time. Clin Psychol Sci. (2018) 6:3–17. 10.1177/2167702617723376

[28] Escobar-Viera CG Shensa A Bowman ND Sidani JE Knight J James AE. Passive and Active Social Media Use and Depressive Symptoms among United States Adults. Cyberpsychol Behav Soc Netw. (2018) 21:437–43. 10.1089/cyber.2017.0668 29995530

[29] Shensa A Escobar-Viera CG Sidani JE Bowman ND Marshal MP Primack BA. Problematic social media use and depressive symptoms among U.S. young adults: a nationally-representative study. Soc Sci Med. (2017) 182:150–7. 10.1016/j.socscimed.2017.03.061 28446367 PMC5476225

[30] Shensa A Sidani JE Dew MA Escobar-Viera CG Primack BA. Social media use and depression and anxiety symptoms: a cluster analysis. Am J Health Behav. (2018) 42:116–28. 10.5993/AJHB.42.2.11 29458520 PMC5904786

[31] Matar Boumosleh J Jaalouk D. Depression, anxiety, and smartphone addiction in University students- a cross sectional study. PLoS ONE. (2017) 12:e0182239. 10.1371/journal.pone.0182239 28777828 PMC5544206

[32] Yang J Fu X Liao X Li Y. Association of problematic smartphone use with poor sleep quality, depression, and anxiety: a systematic review and meta-analysis. Psychiatry Research. (2020) 284:112686. 10.1016/j.psychres.2019.112686 31757638

[33] Ferrari AJ Somerville AJ Baxter AJ Norman R Patten SB Vos T. Global variation in the prevalence and incidence of major depressive disorder: a systematic review of the epidemiological literature. Psychol Med. (2013) 43:471–81. 10.1017/S0033291712001511 22831756

[34] Whiteford HA Degenhardt L Rehm J Baxter AJ Ferrari AJ Erskine HE. Global burden of disease attributable to mental and substance use disorders: findings from the Global Burden of Disease Study (2010). Lancet. (2013) 382:1575–86. 10.1016/S0140-6736(13)61611-6 23993280

[35] Razzak HA Harbi A Ahli S. Depression: prevalence and associated risk factors in the United Arab Emirates. Oman Med J. (2019) 34:274–83. 10.5001/omj.2019.56 31360314 PMC6642715

[36] Abdel Wahed WY Hassan SK. Prevalence and associated factors of stress, anxiety and depression among medical Fayoum University students. Alexandria J Med. (2017) 53:77–84. 10.1016/j.ajme.2016.01.005

[37] Al-Busaidi Z Bhargava K Al-Ismaily A Al-Lawati H Al-Kindi R Al-Shafaee M. Prevalence of depressive symptoms among University students in Oman. Oman Med J. (2011) 26:235–9. 10.5001/omj.2011.58 22043426 PMC3191716

[38] Hamdan-Mansour AM Halabi JO Dawani HA. Depression, hostility, and substance use among University students in Jordan. Ment Heal Subst Use Dual Diagnosis. (2009) 2:52–63. 10.1080/17523280802593301

[39] Malak MZ Khalifeh AH. Anxiety and depression among school students in Jordan: prevalence, risk factors, and predictors. Perspect Psychiatr Care. (2018) 54:242–50. 10.1111/ppc.12229 28617949

[40] GriffithsMDMamunMA. COVID-19 suicidal behavior among couples and suicide pacts: case study evidence from press reports. Psychiatry Res. (2020) 289:113105. 10.1016/j.psychres.2020.11310533242807

[41] Bhuiyan AKMI Sakib N Pakpour AH Griffiths MD Mamun MA. COVID-19-related suicides in Bangladesh due to lockdown and economic factors: case study evidence from media reports. Int J Mental Health Addiction. (2020) 15:1–6. 10.1007/s11469-020-00307-y 32427168 PMC7228428

[42] Mamun MA Ullah I. COVID-19 suicides in Pakistan, dying off not COVID-19 fear but poverty?–The forthcoming economic challenges for a developing country. Brain Behav Immunity. (2020) 87:163–6. 10.1016/j.bbi.2020.05.028 32407859 PMC7212955

[43] Lei L Huang X Zhang S Yang J Yang L Xu M. Comparison of prevalence and associated factors of anxiety and depression among people affected by versus people unaffected by quarantine during the COVID-19 epidemic in Southwestern China. Med Sci Monit. (2020) 26:e924609–1. 10.12659/MSM.924609 32335579 PMC7199435

[44] Ozamiz-Etxebarria N Dosil-Santamaria M Picaza-Gorrochategui M Idoiaga-Mondragon N. Stress, anxiety, and depression levels in the initial stage of the COVID-19 outbreak in a population sample in the northern Spain. Cad Saude Publica. (2020) 36:1–9. 10.1590/0102-311x00054020 32374806

[45] Liu X Kakade M Fuller CJ Fan B Fang Y Kong J. Depression after exposure to stressful events: lessons learned from the severe acute respiratory syndrome epidemic. Compr Psychiatry. (2012) 53:15–23. 10.1016/j.comppsych.2011.02.003 21489421 PMC3176950

[46] Buysse DJ Reynolds CF Monk TH Berman SR Kupfer DJ. The Pittsburgh sleep quality index: a new instrument for psychiatric practice and research. Psychiatry Res. (1989) 28:193–213. 10.1016/0165-1781(89)90047-4 2748771

[47] Radloff LS. The CES-D scale: a self-report depression scale for research in the general population. Appl Psychol Meas. (1977) 1:385–401. 10.1177/014662167700100306

[48] The Hashemite Kingdom of Jordan, The National Council For Family Affairs 2017 survey results. (2018).

[49] WHO | World Health Organization. Available online at: https://www.who.int/ (accessed July 23, 2020).

[50] WHO | World Health Organization [Internet]. Available online at: http://gamapserver.who.int/gho/interactive_charts/tobacco/use/atlas.html (accessed July 25, 2020).

[51] Lemma S Berhane Y Worku A Gelaye B Williams MA. Good quality sleep is associated with better academic performance among University students in Ethiopia. Sleep Breath. (2014) 18:257–63. 10.1007/s11325-013-0874-8 23928956 PMC3918486

[52] Zhang B Wing YK. Sex differences in insomnia: a meta-analysis. Sleep. (2006) 29:85–93. 10.1093/sleep/29.1.85 16453985

[53] Dong X Wang Y Chen Y Wang X Zhu J Wang N. Poor sleep quality and influencing factors among rural adults in Deqing, China. Sleep Breath. (2018) 22:1213–20. 10.1007/s11325-018-1685-8 29936592

[54] Alqarni AB Alzahrani NJ Alsofyani MA. The interaction between sleep quality and academic performance among the medical students in taif University. Egypt J Hosp Med. (2018) 70:2202–8. 10.12816/0045053

[55] Al Shammari MA Al Amer NA Al Mulhim SN Al Mohammedsaleh HN Alomar RS. The quality of sleep and daytime sleepiness and their association with academic achievement of medical students in the eastern province of Saudi Arabia. J Fam Community Med. (2020) 27:97–102. 10.4103/jfcm.JFCM_160_19 32831554 PMC7415273

[56] Jalil El hangouche A Jniene A Aboudrar souad Errguig leila rkain hanan cherti M. Advances in medical education and practice dovepress relationship between poor quality sleep, excessive daytime sleepiness and low academic performance in medical students. Adv Med Educ Pract. (2018) 9–631. 10.2147/AMEP.S162350 30233270 PMC6135210

[57] Nair US Haynes P Collins BN. Baseline sleep quality is a significant predictor of quit-day smoking self-efficacy among low-income treatment-seeking smokers. J Health Psychol. (2019) 24:1484–93. 10.1177/1359105317740619 29139311

[58] Riedel BW Durrence HH Lichstein KL Taylor DJ Bush AJ. The relation between smoking and sleep: the influence of smoking level, health, and psychological variables. Behav Sleep Med. (2004) 2:63–78. 10.1207/s15402010bsm0201_6 15600225

[59] Purani H Friedrichsen S Allen AM. Sleep quality in cigarette smokers: associations with smoking-related outcomes and exercise. Addict Behav. (2019) 90:71–6. 10.1016/j.addbeh.2018.10.023 30368021 PMC6324958

[60] Liao Y Xie L Chen X Kelly BC Qi C Pan C. Sleep quality in cigarette smokers and nonsmokers: findings from the general population in central China. BMC Public Health. (2019) 19. 10.1186/s12889-019-6929-4 31234809 PMC6591832

[61] Khan NA Yogeswaran S Wang Q Muthumalage T Sundar IK Rahman I. Waterpipe smoke and e-cigarette vapor differentially affect circadian molecular clock gene expression in mouse lungs. Khan MF, editor. PLoS ONE. (2019) 14:e0211645. 10.1371/journal.pone.0211645 30811401 PMC6392409

[62] Hwang JW Sundar IK Yao H Sellix MT Rahman I. Circadian clock function is disrupted by environmental tobacco/cigarette smoke, leading to lung inflammation and injury via a SIRT1-BMAL1 pathway. FASEB J. (2014) 28:176–94. 10.1096/fj.13-232629 24025728 PMC3868829

[63] Grandner MA Patel NP Gehrman PR Xie D Sha D Weaver T. Who gets the best sleep? Ethnic and socioeconomic factors related to sleep complaints. Sleep Med. (2010) May;11:470–8. 10.1016/j.sleep.2009.10.006 20388566 PMC2861987

[64] Anders MP Breckenkamp J Blettner M Schlehofer B Berg-Beckhoff G. Association between socioeconomic factors and sleep quality in an urban population-based sample in Germany. Eur J Public Health. (2014) 24:968–73. 10.1093/eurpub/ckt175 24280873

[65] Arcuri J. Students with medical-related majors more likely to have poor quality sleep [Internet]. Available online at: http://www.health.am/sleep/more/students-have-poor-quality-sleep/ (accessed August 31, 2020).

[66] Ayala EE Berry R Winseman JS Mason HRC. A cross-sectional snapshot of sleep quality and quantity among US medical students. Acad Psychiatry. (2017) 41:664–8. 10.1007/s40596-016-0653-5 28091977

[67] Chen B Liu F Ding S Ying X Wang L Wen Y. Gender differences in factors associated with smartphone addiction: a cross-sectional study among medical college students. BMC Psychiatry. (2017) 17:341. 10.1186/s12888-017-1503-z 29017482 PMC5634822

[68] Kuehner C. Gender differences in unipolar depression: an update of epidemiological findings and possible explanations. Acta Psychiatr Scand. (2003) 108:163–74. 10.1034/j.1600-0447.2003.00204.x 12890270

[69] Naninck EFG Lucassen PJ Bakker J. Sex differences in adolescent depression: do sex hormones determine vulnerability? J Neuroendocrinol. (2011) 23:383–92. 10.1111/j.1365-2826.2011.02125.x 21418338

[70] Low-Income Communities _ Anxiety and Depression Association of America, ADAA.

[71] Chen L Wang L Qiu XH Yang XX Qiao ZX Yang YJ. Depression among Chinese University Students: prevalence and socio-demographic correlates. PLoS ONE. (2013) 8:1–6. 10.1371/journal.pone.0058379 23516468 PMC3596366

[72] Hawryluck L Gold WL Robinson S Pogorski S Galea S Styra R. SARS control and psychological effects of quarantine, Toronto, Canada. Emerg Infect Dis. (2004) 10:1206–12. 10.3201/eid1007.030703 15324539 PMC3323345

[73] Tang W Hu T Hu B Jin C Wang G Xie C. Prevalence and correlates of PTSD and depressive symptoms one month after the outbreak of the COVID-19 epidemic in a sample of home-quarantined Chinese University students. J Affect Disord. (2020) 274:1–7. 10.1016/j.jad.2020.05.009 32405111 PMC7217769

[74] Lao XQ Liu X Deng H-B Chan T-C Ho KF Wang F. sleep quality, sleep duration, and the risk of coronary heart disease: a prospective cohort study with 60,586 adults. J Clin sleep Med JCSM Off Publ Am Acad Sleep Med. (2018) 14:109–17. 10.5664/jcsm.6894 29198294 PMC5734879

[75] Yamamoto R Shinzawa M Isaka Y Yamakoshi E Imai E Ohashi Y. Sleep quality and sleep duration with CKD are associated with progression to ESKD. Clin J Am Soc Nephrol. (2018) 13:1825–32. 10.2215/CJN.01340118 30442866 PMC6302324

[76] Okun ML Mancuso RA Hobel CJ Schetter CD Coussons-Read M. Poor sleep quality increases symptoms of depression and anxiety in postpartum women. J Behav Med. (2018) 41:703–10. 10.1007/s10865-018-9950-7 30030650 PMC6192841

[77] Yu J Rawtaer I Fam J Jiang M-J Feng L Kua EH. Sleep correlates of depression and anxiety in an elderly Asian population. Psychogeriatrics. (2016) 16:191–5. 10.1111/psyg.12138 26179204

[78] Kalmbach DA Arnedt JT Song PX Guille C Sen S. Sleep disturbance and short sleep as risk factors for depression and perceived medical errors in first-year residents. Sleep. (2017) 40:1–8. 10.1093/sleep/zsw073 28369654 PMC6084763

[79] Becker NB Jesus SN João KADR Viseu JN Martins RIS. Depression and sleep quality in older adults: a meta-analysis. Psychol Health Med. (2017) 22:889–95. 10.1080/13548506.2016.1274042 28013552

[80] Wang X Cheng S Xu H. Systematic review and meta-analysis of the relationship between sleep disorders and suicidal behaviour in patients with depression. BMC Psychiatry. (2019) 19:303. 10.1186/s12888-019-2302-5 31623600 PMC6798511

[81] Çelik N Ceylan B Ünsal A Çagan Ö. Depression in health college students: relationship factors and sleep quality. Psychol Health Med. (2019) 24:625–30. 10.1080/13548506.2018.1546881 30463430

[82] Gee B Orchard F Clarke E Joy A Clarke T Reynolds S. The effect of non-pharmacological sleep interventions on depression symptoms: a meta-analysis of randomised controlled trials. Sleep Med Rev. (2019) 43:118–28. 10.1016/j.smrv.2018.09.004 30579141

[83] Dalky HF Gharaibeh A. Depression, anxiety, and stress among college students in Jordan and their need for mental health services. Nurs Forum. (2019) 54:205–12. 10.1111/nuf.12316 30554406

